# Genomic Investigation of a Legionellosis Outbreak in a Persistently Colonized Hotel

**DOI:** 10.3389/fmicb.2015.01556

**Published:** 2016-01-21

**Authors:** Leonor Sánchez-Busó, Silvia Guiral, Sebastián Crespi, Víctor Moya, María L. Camaró, María P. Olmos, Francisco Adrián, Vicente Morera, Francisco González-Morán, Hermelinda Vanaclocha, Fernando González-Candelas

**Affiliations:** ^1^Unidad Mixta “Infección y Salud Pública” Fundación para el Fomento de la Investigación Sanitaria y Biomédica de la Comunitat Valenciana-Salud Pública, Universitat de ValènciaValencia, Spain; ^2^Centros de Investigación Biomédica en Red Epidemiología y Salud PúblicaValencia, Spain; ^3^Dirección General de Salud Pública, Subdirección de Epidemiología y Vigilancia de la SaludValencia, Spain; ^4^Biolinea IntPalma de Mallorca, Spain; ^5^Policlínica MiramarPalma de Mallorca, Spain; ^6^LegioprevValencia, Spain; ^7^Dirección General de Salud Pública, Laboratorio de Salud PúblicaValencia, Spain; ^8^Centro de Salud Pública DeniaDenia, Spain

**Keywords:** Legionnaire's disease (LD), outbreaks, typing, complete genome, mixed infections

## Abstract

**Objectives:** A long-lasting legionellosis outbreak was reported between November 2011 and July 2012 in a hotel in Calpe (Spain) affecting 44 patients including six deaths. Intensive epidemiological and microbiological investigations were performed in order to detect the reservoirs.

**Methods:** Clinical and environmental samples were tested for the presence and genetic characterization of *Legionella pneumophila*. Six of the isolates were subjected to whole-genome sequencing.

**Results:** Sequencing of 14 clinical and 260 environmental samples revealed sequence type (ST) 23 as the main responsible strain for the infections. This ST was found in the spa pool, from where it spread to other hotel public spaces, explaining the ST23 clinical cases, including guests who had not visited the spa. Uncultured clinical specimens showed profiles compatible with ST23, ST578, and mixed patterns. Profiles compatible with ST578 were obtained by direct sequencing from biofilm samples collected from the domestic water system, which provided evidence for the source of infection for non ST23 patients. Whole genome data from five ST23 strains and the identification of different STs and *Legionella* species showed that different hotel premises were likely colonized since the hotel opening thus explaining how different patients had been infected by distinct STs.

**Conclusions:** Both epidemiological and molecular data are essential in the investigation of legionellosis outbreaks. Whole-genome sequencing data revealed significant intra-ST variability and allowed to make further inference on the short-term evolution of a local colonization of *L. pneumophila*.

## Introduction

*Legionella* infections are opportunistic and the inhalation of aerosols with enough bacterial loads can cause a severe form of pneumonia, known as Legionnaires' disease (Fraser et al., 1977), or a milder flu-like condition, denoted as Pontiac fever (Glick et al., 1978). After its first identification in 1976 in a Legionnaires' convention in a hotel in Philadelphia, many legionellosis outbreaks have involved travel-associated clusters (Benin et al., 2002; Burnsed et al., 2007; Rota et al., 2011).

Legionellosis outbreaks are usually studied by typing pure *Legionella* cultures from infected patients with different molecular techniques, assuming that only one strain is causing the disease. However, co-infection with different *Legionella* species has also been reported (Meyer et al., 1980; Muder et al., 1983; Buchbinder et al., 2004; Matsui et al., 2010; McAdam et al., 2014; Wewalka et al., 2014), as well as with different serogroups of *L. pneumophila* (Meyer et al., 1980; Yiallouros et al., 2013; Wewalka et al., 2014). The introduction of new typing methods based on direct amplification and sequencing of *Legionella* from clinical samples (Coscollá and González-Candelas, 2009; Ginevra et al., 2009; Mentasti et al., 2012) has also revealed that outbreak patients can be infected simultaneously by more than one *Legionella* strain (Coscollá et al., 2014), even from the same serogroup. Dual or multiple infections pose an additional difficulty for the identification and subsequent control of outbreak sources.

In November 2011, the first cases of a legionellosis outbreak that lasted 33 weeks were reported in a hotel in the locality of Calpe (Comunidad Valenciana, Spain). The outbreak comprised four different clusters with two suspected and 42 confirmed patients that matched the European case definition including six fatalities. The hotel had a previous history of six legionellosis cases in 2006, just 3 months after it was opened to the public and one case in 2007. However, no additional cases had been reported until the outbreak analyzed in this work, in which both tourists and workers were affected.

Upon notification of the first cases, public health officials started an environmental investigation and the implementation of control measures (Vanaclocha et al., 2012). These included the disinfection of the water distribution system of the hotel and its closure. Here, we present the molecular analyses performed during the investigation of this outbreak using both cultured and uncultured clinical and environmental samples that helped unveil the source and route of infection for all the patients. Whole-genome sequencing (WGS) was performed for some of the isolates to get higher discrimination than the traditional DNA Sequence-Based Typing (SBT) and to infer when *Legionella pneumophila* might have colonized the facility.

## Materials and methods

### Case definition and epidemiological description of the outbreak

Confirmed cases were defined as patients who, having stayed or worked at the hotel between 2 and 10 days before the onset of symptoms, showed a clinical diagnosis of pneumonia with laboratory findings which confirmed infection by *Legionella*, including a positive urine test for *L. pneumophila* antigen or a positive culture isolation of the bacteria from respiratory secretions.

**Figure 1:**
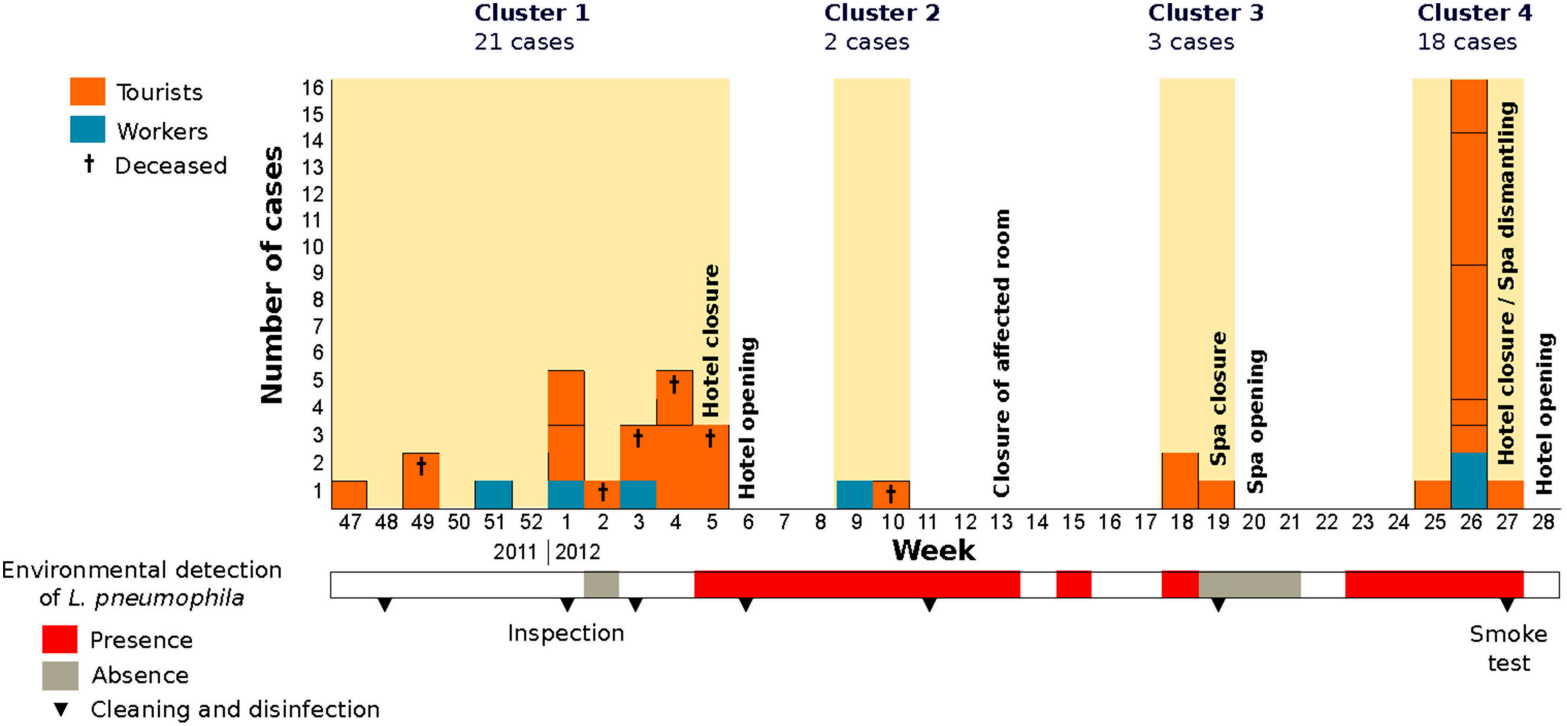
**Number of cases affected by legionellosis grouped per week of onset of symptoms**. Detection of *L. pneumophila* during the environmental investigation is indicated by colors in the lower bar (see legend). Measures undergone by local health authorities are also marked in the corresponding weeks.

The outbreak was epidemiologically divided into four temporal clusters that involved 44 cases, 38 tourists (average age 71.5) and six hotel workers (average age 49.5) (Figure 1). Visitors had stayed at 28 different rooms in the hotel, distributed in 11 floors. Six of the rooms accommodated two cases and other three patients had also used the same room, two of them simultaneously. The outbreak involved six deaths (average age 77.2), and deceased patients had stayed at five different rooms: two of those patients had stayed in the same room at different times. Only five tourists and two workers had ever been in the facility of the local spa, which is located below the main hall from where it is easily visible through a glass dome.

### Clinical samples

Clinical samples from 14 outbreak patients were received for study in our local reference laboratory for *L. pneumophila* infections. Specifically, eight sputum, two broncho-alveolar aspirates (BAS), one lung puncture aspirate and three cultures obtained in the Microbiology Service of the corresponding hospitals, two of them from autopsies, were collected and transported at 4°C for genetic testing. Total DNA was extracted from uncultured clinical samples using UltraClean® BloodSpin® DNA Isolation Kit (MoBio). DNA from the three isolates was extracted using a thermal shock as described below for environmental isolates.

### Environmental sampling

A total of 632 two-liter water samples and 164 biofilm swabs (per duplicate) were taken during the environmental investigation of the outbreak from the different water systems of the hotel including the spa pool. Water samples were tested for biocides, pH and temperature. Pieces of air filters of the air-conditioning system connecting the spa and the hall area were also collected for testing. All samples were transported immediately to the lab for *L. pneumophila* detection and genetic characterization.

Water filtering and culturing were performed following the AFNOR NF T 90-431 2 (2003) and UNE-EN ISO 11731-2:2008 regulations on water quality and standard culture-based detection of *Legionella* spp. Isolates were serogrouped using Oxoid *Legionella* Latex Test (Thermo Scientific), subtyped using monoclonal antibodies (Joly et al., 1983, 1986), and subsequently characterized genetically. Different colonies from the culture plates were analyzed, when available, to check for strains of different genetic profile.

Biofilm swabs and air filters were submerged into lysis buffer (100 mM Tris HCl, 100 mM NaCl, 1 mM EDTA, 10% SDS and ultrapure water, final pH 8.1) and incubated with 1 mg/mL proteinase K during 10 min at 65°C for biofilm swabs and 30 min at the same temperature for the other samples. Cotton swabs were introduced into 0.7 mL microtubes with an opening at the bottom placed inside a 2-mL microtube (Suenaga and Nakamura, 2005) and centrifuged at maximum speed for 10 min to recover the maximum amount of lysate. Final retrieved volumes were used for direct DNA purification using the spin filter protocol of the UltraClean® BloodSpin® DNA Isolation Kit (MoBio).

DNA from *L. pneumophila* culture isolates was extracted by thermal shock. A small amount of bacteria was suspended in 200 μL of autoclaved, ultrapure water and subjected to two cycles of a 5-min incubation step at 99°C followed by 5 min at 4°C. Cell debris was pelleted by centrifuging during 3 min at 12,000 rpm, and the DNA-containing supernatant was used for molecular amplification.

### *L. pneumophila* detection and sequence-based typing (SBT)

Touchdown PCR (TD-PCR; Don et al., 1991, targeting two regions of the Sequence-Based Typing (SBT), Gaia et al., 2005; Farhat et al., 2011) scheme (*pilE* and *asd*) was performed on the DNA samples extracted from biofilms for *L. pneumophila* detection. Positive samples were subsequently used for amplifying the five remaining loci (*fliC, mip, mompS, proA*, and *neuA*) in the SBT scheme for this species. Amplification was performed using 2.5 U FastStart High Fidelity PCR System (Roche Applied Science), 1X Buffer with MgCl2 1.8 mM, 200 μM of each dNTP, 4% DMSO, 0.2 μM of the corresponding pair of primers, an amount of DNA suspension depending on its concentration (measured three times by NanoDrop 1000TM, Thermo Scientific) and autoclaved and filtered ultrapure water to a final volume of 25 μL. The thermal profile of the TD-PCR included an initial denaturation step of 5 min at 95°C followed by 10 cycles of denaturation (30 s at 95°C), annealing (30 s at a primer-dependent temperature, Sánchez-Busó et al., 2015) and at a ramp rate of −1°C/cycle) and extension (30 s at 72°C). Immediately after the TD step, 35 cycles of standard PCR were applied using the same conditions but fixing the annealing temperature at the optimal Tm for each region; a final extension step of 8 min at 72°C was also used to ensure complete amplification.

DNA retrieved from *L. pneumophila* pure cultures and uncultured clinical samples from confirmed patients were used for amplification of the 7 loci in the SBT scheme by traditional (Gaia et al., 2003, 2005; Coscollá and González-Candelas, 2007) and seminested PCR amplification (Coscollá and González-Candelas, 2009). PCR products were purified and Sanger-sequenced as previously described in Coscollá and González-Candelas (2007, 2009).

### Cloning of PCR products

PCR products from five positive biofilm samples obtained in hotel rooms associated to clinical cases and that showed evidence of multiple *L. pneumophila* variants in *fliC, pilE*, and *asd* (**Table 2**) were cloned into *Escherichia coli* JM109 Competent Cells (Promega) using pGEM®-T Easy vector System II (Promega). In order to check whether the minor variants detected could be considered actual polymorphisms and not artifacts resulting from the amplification and/or cloning process, TD-PCR products from pure cultures of these three regions were cloned and sequenced. PCR products from the same regions obtained from four sputum/BAS samples were also cloned (**Table 2**) in order to check for intra-patient variability. Ligation and transformation reactions were performed following the instructions of the manufacturer.

### Phylogenetic reconstruction of SBT data

The sequences of the alleles involved in the STs found in the analyzed samples were downloaded from the EWGLI SBT database (http://www.hpa-bioinformatics.org.uk/legionella/legionella_sbt/php/sbt_homepage.php) and concatenated in order to create a haplotype sequence for each strain. Complete and incomplete profiles (with at least two sequenced loci) were used for maximum likelihood phylogenetic reconstruction using RAxML v7.2.8 (Stamatakis, 2006) with the GTRGAMMA model of substitution and 1000 bootstrap replicates along with reference STs.

### Whole-genome sequencing on outbreak isolates

Four clinical ST23 and 2 environmental isolates (ST23 and ST1236) were selected because of their DNA quality and quantity for whole-genome sequencing using the SOLiD 5500XL platform. Single-end reads of 75 bp with an average per nucleotide coverage of 90X were mapped against the assembly of one of the ST23 strains (VelvetOptimiser v2.2.5, Zerbino, 2010), ID_125 (resulting scaffold of 3307, 11 bp), following the pipeline described in Sánchez-Busó et al. (2014). Filtered SNPs from the core genome of the six strains and nine reference genomes from GenBank [Paris—NC_006368 (Cazalet et al., 2004), Lens—NC_006369 (Cazalet et al., 2004), Philadelphia1—NC_002942 (Chien et al., 2004), Alcoy—NC_014125 (D'Auria et al., 2010), Corby—NC_009494 (Steinert et al., 2007), Lorraine—NC_018139 (Gomez-Valero et al., 2011)], 130b—FR687201 (Schroeder et al., 2010), ATCC43290—NC_016811 and HL06041035—NC_018140) were used for phylogenetic reconstruction under the minimum evolution criterion (Rzhetsky and Nei, 1993). To do so, pairwise distances and 1000 bootstrap replicates were applied with MEGA5 (Tamura et al., 2011). SOLiD genomic data have been deposited in the European Nucleotide Archive (ENA) under project accession PRJEB5990.

### Bayesian estimation of colonization time

In order to study the time at which ST23 could have started proliferating within the hotel, we used the ST23 non-homoplastic core alignment (3,267,949 bp) for Bayesian estimation with BEAST v1.7.5 (Drummond et al., 2012). The HKY model of nucleotide substitution and an uncorrelated lognormal molecular clock were selected using the substitution rate previously estimated for ST578 (Sánchez-Busó et al., 2014) and the isolation dates as priors. Four parallel Markov chains using the same parameters were run for 10,000,000 generations with samples taken every 10,000 steps. Convergence of the chains was tested with Tracer v1.5 when the ESS for all the parameters was >200.

## Results

### Clinical *L. pneumophila* SBT profiles

*L. pneumophila* could be cultured from only four (28.57%) of 14 the clinical samples studied in our laboratory, two from pulmonary tissue retrieved during autopsy and the other two from sputums. All of them were typed as ST23 (Table 1), in agreement with the results obtained by Public Health England (PHE) from the first travel-associated cases related to the hotel that were reported after returning to their country of origin (Vanaclocha et al., 2012). Eleven uncultured clinical samples (one of them was also culture-positive) were analyzed by direct amplification and sequencing as described. Three of them provided a complete genetic profile (ST23) and an additional sample (163655) resulted in a new profile that differed from ST23 in two loci (*fliC* and *mompS*; Table 1). Other two samples (191712 and 192147) presented very divergent profiles from ST23. However, some of the sequencing reads from these and two other samples (192061 and 163655) presented unresolved nucleotides at several positions, which led us to seek determining their sequence with better precision.

**Table 1:** **Sequence-based Typing of *L. pneumophila* strains from clinical samples**.

**Sample**	**Type of sample**	***fliC***	***pilE***	***asd***	***mip***	***mompS***	***proA***	***neuA***	**ST**
125	Culture	2	3	9	10	2	1	6	23
191712	Sputum	**6**	**10**	**1,15**	13	–	14	6	(578)
192091	Culture	2	3	9	10	2	1	6	23
	Sputum	–	**3**	**9**	10	2	1	6	23
163655	Sputum	**6**	**3**	**9**	10	9	1	6	A^*^
192147	Sputum	**6**	**10**	**1**	13	2	–	6	–
50291	Culture	2	3	9	10	2	1	6	23
50726	Culture	2	3	9	10	2	1	6	23
191620	Lung puncture	2	3	9	10	2	1	6	23
193975	Sputum	6	3	–	–	2	1	6	–
9138	Sputum	–	–	9	–	–	–	–	–
160613	Sputum	2	3	9	10	2	1	6	23
18484	Sputum	2	3	9	–	2	1	6	–
160717	BAS	2	3	9	10	2	1	6	23

*Shadowed bold numbers correspond to PCR products that were cloned for further genetic testing. BAS, Broncho-alveolar aspirate*.

* *ST-A corresponds to a new profile that could not be assigned a ESGLI profile because the results were derived from an uncultured sample*.

Cloning and sequencing of PCR products from three loci (*fliC, pilE*, and *asd*; Table 2) from the 4 uncultured sputums detailed above revealed the presence of two variants (*asd*-1 and *asd*-15) in patient 191712, providing evidence for the presence of an infecting strain that matched that found in patient 192147. It is worth mentioning that these two patients were a couple who had stayed in the same hotel room and that although the corresponding profiles missed one locus each, their combination corresponded to ST578 (Table 1).

**Table 2:** **List of clinical and environmental biofilm samples subjected to cloning of PCR products**.

**Sample**	**C/E^a^**	**Allele pattern^b^**	**ST**	**Implication**
191712	C	**6,10,1/15**,13,X,14,6	578^c^	Patient
192147	C	**6,10,1**,13,2,X,6	–	Patient
163655	C	**6,3,9**,10,9,1,6	B	Patient
192091	C	**X,3,9**,10,2,1,6	23^c^	Patient
cal12_18	E	**+,+,+**,+,+,+,+	+	Hotel room with 2 cases (1 death)
cal12_40	E	**6,10,15**,X,9,14,6	578^c^	Hotel room with 1 case (1 death)
cal12_46	E	**6,10,15**,13,9,14,6	578	Hotel room with 2 cases (1 death)
cal12_97	E	**6,10,+**,+,9,14,+	578^c^	Hotel room with 1 case
cal12_151	E	**6,10,15**,13,9,14,6	578	Hotel room with 2 cases (2 deaths)
F3	E	**2/6,3,9**,10,2,1,6	23	Air filter

*Underlined bold regions were used (fliC, pilE, asd, mip, mompS, proA, neuA). Selected biofilm samples were taken from different rooms*.

a *C, clinical; E, environmental*.

b *X, PCR amplification failed; +, positive amplification but allele assignation failed*.

c *Incomplete allele pattern but compatible with ST23 or ST578*.

### Environmental *L. pneumophila* SBT profiles

From the sampled biofilms, 110 (67.1%) resulted positive for at least one of the two SBT loci initially tested, *pilE* and *asd*. PCR and further sequencing of the remaining other loci revealed that a profile compatible with ST578 was frequently found (28.2%, 31/110) in samples from most of the rooms studied and other common hotel facilities (Table 3). Two additional STs, ST1136, and ST23, could be identified by SBT from the uncultured samples, with two and one representatives respectively. These two STs differ in only one nucleotide in locus *fliC*. Only one biofilm swab was taken from the spa at the end of January 2012 and it tested negative.

**Table 3:** **Results of the molecular investigation of environmental samples**.

**Date**	**Sample**	**Location**	***N***	**Type of sample**	***fliC***	***pilE***	***asd***	***mip***	***mompS***	***proA***	***neuA***	**ST/Species**	**Serogroup/MAb type**
Jan-March 2012	Several	Hotel rooms, hall, restrooms	5	Biofilm	6	10	15	13	9	14	6	578	–
Jan-March 2012	Several	Hotel rooms, hall, restrooms	26	Biofilm	(6)	(10)	(15)	(13)	(9)	(14)	(6)	(578) (at least 5 loci)	–
Jan-March	Several	Hotel rooms, hall, restrooms	74	Biofilm	+	+	+	+	+	+	+	Incomplete STs (< 5 loci)	–
07/07/2012	PF2	Air filter	1	Biofilm	6	3	–	–	–	–	–	–	–
07/07/2012	PF3	Air filter	1	Biofilm	–	3	9	–	–	1	6	–	–
07/07/2012	F1	Air filter	1	Biofilm	6	3	9	10	2	1	6	1164	–
07/07/2012	F2	Air filter	1	Biofilm	6	3	9	10	2	1	6	1164	–
07/07/2012	F3	Air filter	1	Biofilm	2	3	9	10	2	1	6	23	–
31/01/2012	918	Spa pool	2	Culture	6	10	15	10	21	40	6	1236	1
11/02/2012	12392	Spa pool	1	Culture	2	3	9	10	2	1	6	23	1
23/02/2012	2084	Spa pool	1	Culture	–	–	–	–	–	–	–	*L. micdadei*	–
02/05/2012	4855	Spa pool	9	Culture	–	–	–	–	–	–	–	*L. micdadei*	–
02/05/2012	4856	Spa pool	12	Culture	2	3	9	10	2	1	6	23	1
26/06/2012	8298	Room, cold water	9	Culture	–	–	–	–	–	–	–	–	2–15
04/07/2012	Al657	90 m3 water tank	10	Culture	2	3	9	10	2	1	6	23	1
09/07/2012	8965	Osmosis (in)	7	Culture	2	3	9	10	2	1	6	23	1
09/07/2012	8966	Osmosis (out)	5	Culture	2	3	9	10	2	1	6	23	1
09/07/2012	8968	Spa (fountain)	10	Culture	2	3	9	10	2	1	6	23	1
18/07/2012	69135	Spa pool	1	Culture	–	–	–	–	–	–	–	*L. micdadei*	–
18/07/2012	69147	Spa pool	1	Culture	–	–	–	–	–	–	–	*L. micdadei*	–
18/07/2012	69148	Osmosis device	1	Culture	2	3	9	10	2	1	6	23	1 Allentown/France
18/07/2012	69153	Spa pool	1	Culture	2	3	9	10	2	1	6	23	1 Allentown/France
18/07/2012	69183	Spa pool drainer	1	Culture	2	3	9	10	2	1	6	23	1 Allentown/France
27/07/2012	Al763	Watering network (hotel entrance)	1	Culture	5	2	22	10	6	25	203	1358	8
23/08/2012	10636	Water deposit	1	Culture	–	–	–	–	–	–	–	*L. anisa*	
12/04/2012	4029	Spa pool	4	Culture	2	3	9	10	2	1	6	23	1
12/04/2012	4029	Spa pool	1	Culture	6	10	15	10	21	40	6	1236	1
12/04/2012	4029	Spa pool	1	Culture	–	–	–	–	–	–	–	*L. micdadei*	–
02/05/2012	4857	Spa pool	3	Culture	2	3	9	10	2	1	6	23	1 Allentown/France
02/05/2012	4857	Spa pool	1	Culture	6	10	15	10	21	40	6	1236	1 Allentown/France
02/05/2012	4857	Spa pool	1	Culture	5	2	22	10	6	25	203	1358	10
02/05/2012	4857	Spa pool	2	Culture	–	–	–	–	–	–	–	*L. micdadei*	–
31/01/2012	Al47	Spa pool	3	Culture	6	10	15	10	21	40	6	1236	1
31/01/2012	Al47	Spa pool	3	Culture	–	–	–	–	–	–	–	*L. micdadei*	–

*Biofilm samples from hotel rooms, hall and restrooms (n = 164) were analyzed directly as none produced positive cultures. The results of those samples in which L. pneumophila was detected (n = 105) are shown in the first three rows depending on whether they yielded a complete ST, an almost complete ST (at least five alleles determined to be identical to those defining the ST), or an incomplete ST (less than five alleles determined). For some cultures, several colonies were sequenced, as detailed (N when type of sample is “Culture”). The last three cultured samples revealed a complex composition with two or more STs and/or another Legionella species*.

**Figure 2:**
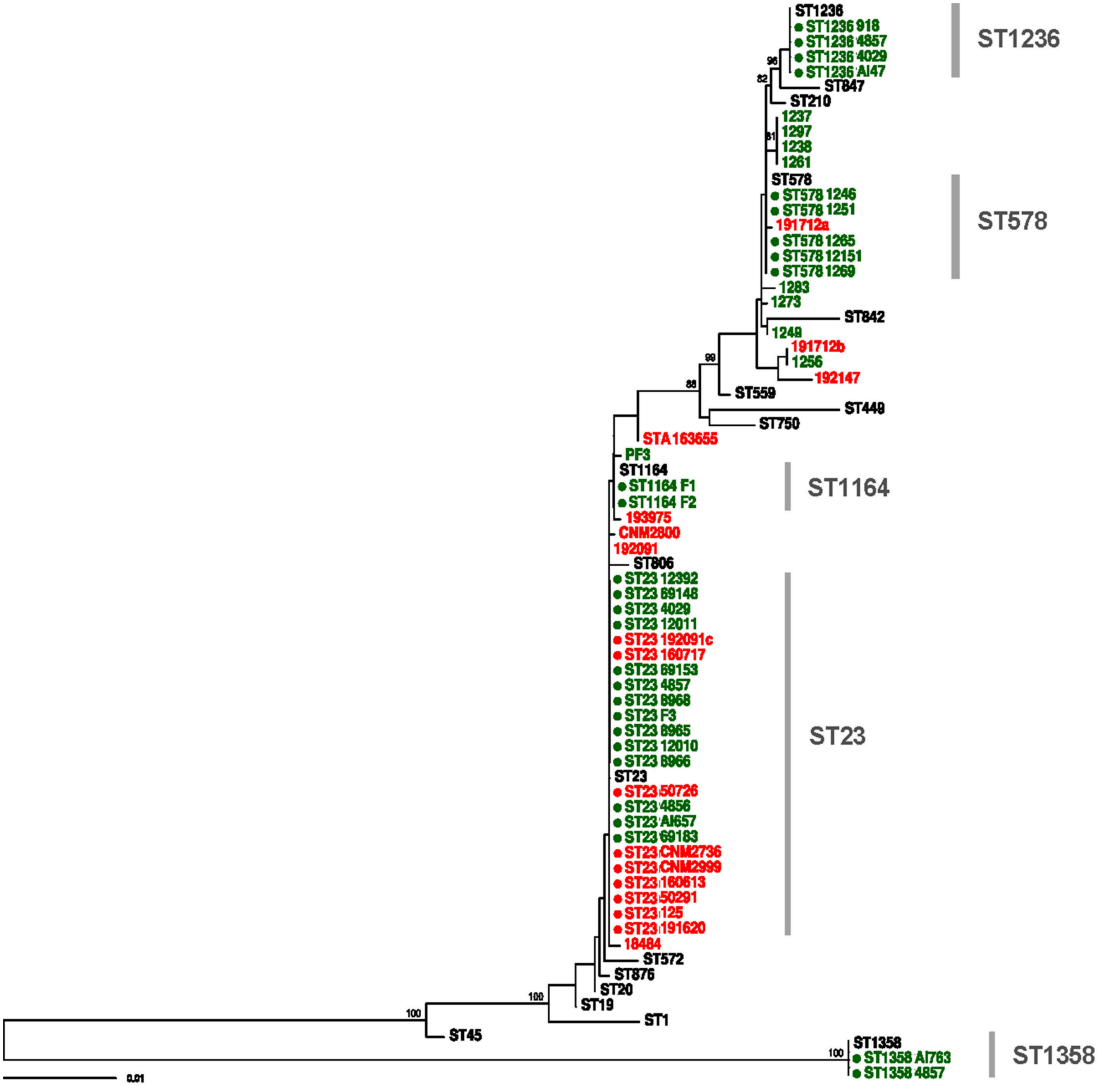
**Maximum likelihood tree constructed with RAxML using SBT data of all clinical and environmental samples included in the study**. Red and green tips represent clinical and environmental samples, respectively. Reference STs are shown in black. Complete profiles are marked with a filled circle. Bootstrap support values higher than 70% are shown.

Twenty positive cultures (3.2%) were obtained from water samples (Table 3), revealing mostly *L. pneumophila* serogroup 1 MAb type Allentown/France ST23 (*n* = 9) and *L. micdadei* (*n* = 4). Two additional STs were identified from these cultured samples, ST1236 and ST1358. ST1236 has a SBT profile which is genetically close to that of ST578, with a difference in only 5 nucleotide sites (Figure 2). In addition, three samples (4039, 4857, and A147) presented a mixture of different *L. pneumophila* STs and *L. micdadei* when several colonies from the same plate were sequenced (Table 3).

The link between the environmental strain ST23 found in the spa pool and most clinical cases was firmly established by the corresponding SBT sequencing results. However, only the direct SBT analysis of uncultured environmental and clinical samples provided evidence for the existence of a second infecting strain found only in the domestic water system, compatible with ST578, which can be linked reliably to at least one clinical case (Tables 1, 2). In general, the phylogenetic tree derived from all these sequence data revealed links between all the clinical cases and environmental samples from the hotel (Figure 2).

Smoke-tracing studies and airflow dynamics models showed that aerosols from the spa area could reach easily the hotel hall by different ways, including the air-conditioning system. Pieces of the air filters collected from the air-handling unit for the hall area were further analyzed by direct SBT. Five of the six filter samples analyzed were positive for two loci of the bacteria (*pilE* and *asd*) by TD-PCR. A mixed pattern was detected for locus *fliC* in one of the samples, which prompted us to use the same procedure for cloning and sequencing of PCR products described previously. The analysis of sequences derived from filter samples revealed the presence of strain ST23, thus providing clear evidence of the presence of this ST in the air-conditioning system of the hotel, and also a compatible profile (ST1164) which differs from ST23 in only one polymorphic site (Tables 2, 3). The sequences obtained from these samples were also included in the phylogenetic tree shown in Figure 2.

### ST23 colonization history analyzed by WGS

Five ST23 isolates (four clinical and one obtained from the spa pool) were subjected to whole-genome sequencing (WGS) to assess the intra-ST23 variability and to gain insight about its colonization history. Additionally, one ST1236 isolate was included to check its relatedness to ST578, from which it is genetically close according to the SBT data (Table 4).

**Table 4:** **Accession numbers to the fastq files of the 6 *L. pneumophila* strains sequenced using SOLiD 5500XL during the outbreak investigation deposited in the European Nucleotide Archive (ENA) under project accession PRJEB5990**.

**Sample**	**Strain**	**Experiment**	**Run**	**ST**	**Source**
125	ERS497776	ERX516043	ERR556974	23	Clinical
192091	ERS497781	ERX516048	ERR556979	23	Clinical
4029	ERS497807	ERX516074	ERR557005	23	Environmental
50291	ERS497828	ERX516095	ERR557026	23	Clinical
50726	ERS497830	ERX516097	ERR557028	23	Clinical
918	ERS497849	ERX516116	ERR557047	1236	Environmental

*Further details on the sequenced samples are given in Tables 1–3*.

**Figure 3:**
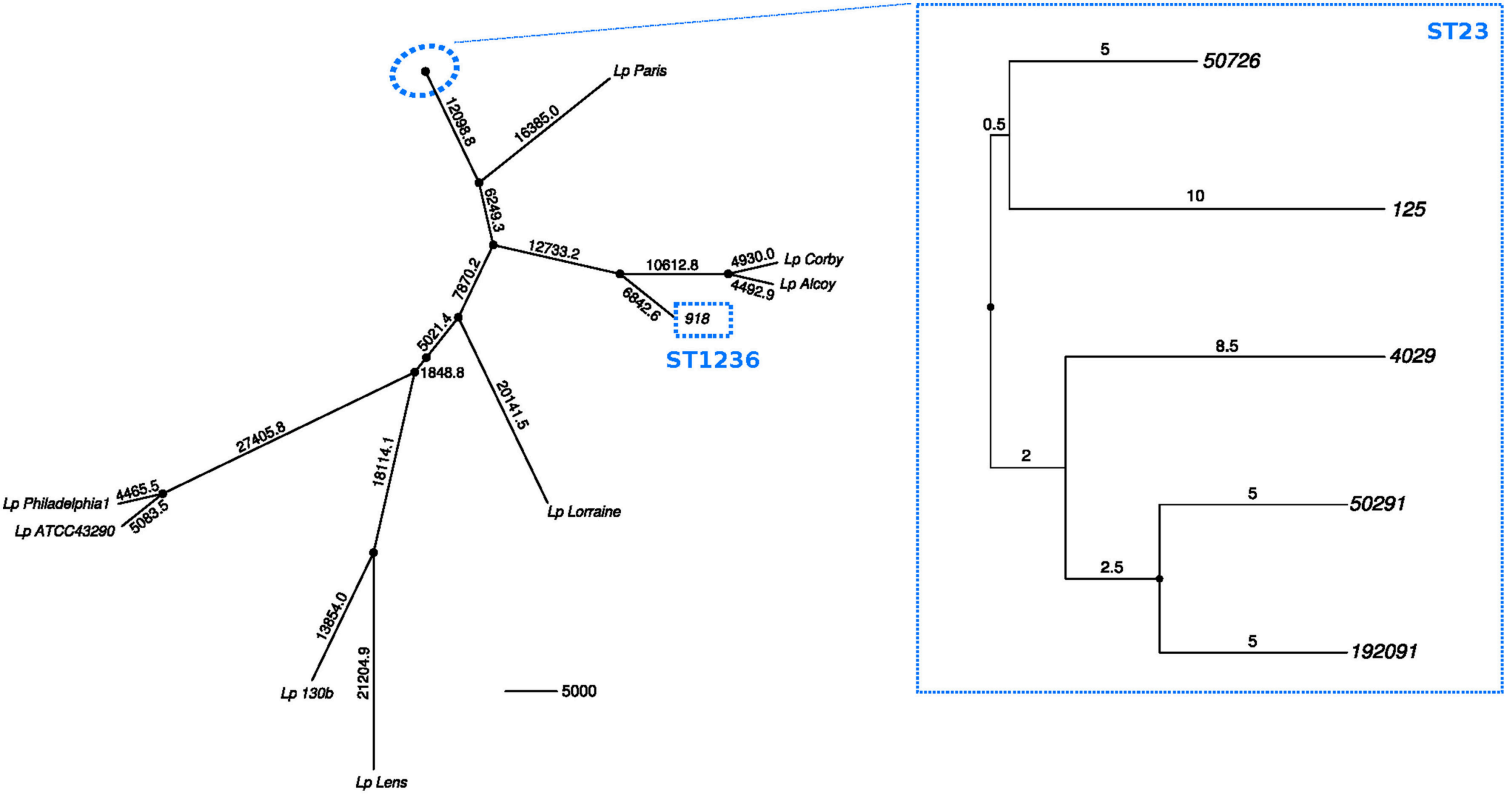
**Minimum-evolution tree constructed using pairwise SNP distances between the sequenced strains and the reference genomes from the databases**. Black dots at nodes represent bootstrap supports higher than 90%. Branch labels indicate number of polymorphisms.

Seventy-one polymorphic sites were detected in the core genome of the five ST23 strains (3,267,949 bp). Twenty-four were estimated as homoplastic and removed from further analyses to account for the effect of recombination, leaving 47 non-homoplastic SNPs. The average number of core SNPs between ST23 isolates was estimated to be 22.4 (range 9–34; 6.85E-06 substitutions per site, s/s) (Figure 3). Strain 918 (ST1236) showed a high divergence from the ST23 strains, with approximately 38,810 SNPs between both STs (core genome size 2,844,379 bp; 0.0136 s/s). SBT data showed just 5 nucleotide differences from ST578 (Figure 2). However, at the genome level, these two strains differ in more than 21,441 positions (0.0083 s/s).

Using a Bayesian inference approach, the substitution rate for the five ST23 strains was estimated to be 7.76E-07 s/s/y (95% Highest Posterior Density (HPD) interval 2.32E-06–2.09E-08), corresponding to an accumulation of approximately 2.5 nucleotide changes in the core genome per year. Hence, these isolates started to diverge from their most recent common ancestor by approximately year 1994 (95% HPD, 1956–2011), much earlier than the hotel opening (2006).

## Discussion

We have described the results of the epidemiological, environmental and genetic investigations of a complex and long-lasting legionellosis outbreak that occurred between November 2011 and July 2012 in a hotel (Calpe, Spain) with 44 cases and six fatal outcomes. During the outbreak, different control measures were adopted, which included repeated cleaning and disinfections of the different water systems of the hotel and two temporary hotel closures (Figure 1). The outbreak was controlled only when the hotel spa pool was permanently closed and completely dismantled. A new spa pool was built with renewed air-conditioning ducts. No new cases of legionellosis related to this hotel have appeared since then.

The initial epidemiological investigation revealed a clear link to the hotel that prompted the first control measures. The first signs of the presence of this bacterium in the hotel were obtained by the direct amplification of two loci from biofilm samples in several hotel rooms only 10 days after the cleaning and disinfection and led to the first closure of the hotel (Vanaclocha et al., 2012). Remarkably, the corresponding water samples were negative for *Legionella* by culture. Repeated samples from the same spots revealed that biofilm-containing *Legionella* recolonized most facilities only a few days after their apparently removal by chlorine treatment.

Most of the sequenced clinical isolates and direct samples corresponded to ST23 whereas a profile compatible with ST578 was repeatedly identified in biofilm samples of the domestic water system. The only exception corresponded to a couple (samples 192712 and 192147) who shared the same room and whose incompletely determined ST differed in five of the six sequenced loci from ST23 but were compatible with ST578 (Table 1). Additional cloning and sequencing of PCR products from these patients revealed the presence of common strains in both of them, thus confirming the existence of a second infecting strain along the outbreak which, in turn, suggested that the domestic water system was another source of infection. Several recent reports (Yiallouros et al., 2013; Coscollá et al., 2014; McAdam et al., 2014; Wewalka et al., 2014) have shown the presence of mixed *Legionella* infections in outbreak patients. Here, we have shown the presence of more than one strain co-existing in the same location and simultaneously producing infections that led to an outbreak.

After the first cluster of cases, ST23 was isolated for the first time in the spa pool, located at the hotel basement. However, the initial epidemiological investigation failed to find a link between many of the patients infected with *L. pneumophila* ST23 and the spa area. In July 2012, a thorough environmental investigation of the spa structure led to the discovery of multiple hidden cavities with stagnant water under the pool vessel that had connections to the bathing water. Subsequent environmental studies suggested the dissemination of ST23 from the spa pool to the hall area through different ways including the air-conditioning system. The identification of *Legionella* profiles compatible with ST23 in the air filters of the air-handling unit for the hall area confirmed the implication of the air-conditioning system in the dissemination of this strain. This helped to explain the detection of this ST in samples from patients who had not visited the spa. Sequencing of isolates from water samples revealed mainly ST23, ST1236, and *Legionella micdadei* as colonizers in different parts of the spa pool, a reverse osmosis plant, a drinking fountain at the spa and a pressure vessel of a non-potable water system.

Genomic data can be used to estimate the evolutionary rates of living organisms (Harris et al., 2010; Bryant et al., 2013; Chewapreecha et al., 2014; Sánchez-Busó et al., 2014), and even the time at which these organisms could have colonized a specific location (Sánchez-Busó et al., 2014). Only three works up to date have used high throughput sequencing to the retrospective study of legionellosis outbreaks (Reuter et al., 2013; Lévesque et al., 2014; McAdam et al., 2014). In the latter case, *L. pneumophila* isolation was possible only from 15/91 patients. In our case, isolation was only successful in 4/14 of the clinical samples. The difficulty in obtaining *L. pneumophila* pure cultures from all the samples is reflected in the subsequent poor representation of the outbreak strains in NGS.

Using Bayesian inference, we estimated the substitution rate of the non-recombinant core genome of the ST23 strains within the hotel as 7.76E-07 s/s/y, higher but of the same order of magnitude than that estimated for the persistent ST578 in the locality of Alcoy (1.39E-07 s/s/y; Sánchez-Busó et al., 2014). The ST23 strains found in the spa were not identical, as would be expected from a single clone colonizing the spa and causing an outbreak. Besides, the time at which ST23 could have started proliferating was estimated to be approximately year 1994. Despite the long HPD intervals from the Bayesian analysis (1956–2011), the finding of 71 polymorphic sites (47 non-homoplastic) reveals a higher diversity than expected. These results, as well as the finding of other STs and *Legionella* species colonizing the hotel, provide evidence that there was a complex community of *L. pneumophila* inhabiting the area previous to the building of the hotel, including a population of ST23. Thus, the hotel could have been colonized by different STs during its construction and before its opening to the public in 2006. Moreover, there was a precedent of legionellosis cases in the period 2006–2007 related to the hotel, but no genetic information about the causing strain was retrieved at that moment, so further comparisons between both clusters cannot be performed.

The results derived from the investigation of this outbreak revealed that information from both cultured and uncultured samples collected during the epidemiological investigation of a complex legionellosis outbreak can be essential for its clarification. Moreover, WGS data from an outbreak investigation can help, not only to the epidemiological investigation in order to detect the main reservoir, but it also allows the application of inference tools to trace back the local evolution of the pathogen. The information retrieved from this type of analysis can be important for public health officials to better understand the local behavior of a pathogen such as *L. pneumophila*, capable of colonizing rapidly several facilities in a complex building and remain silently evolving until conditions favor its multiplication and spread, resulting in the infection of exposed, susceptible persons.

### Conflict of interest statement

The authors declare that the research was conducted in the absence of any commercial or financial relationships that could be construed as a potential conflict of interest.
